# Key Physicochemical Determinants in the Antimicrobial Peptide RiLK1 Promote Amphipathic Structures

**DOI:** 10.3390/ijms221810011

**Published:** 2021-09-16

**Authors:** Lucia Falcigno, Gabriella D’Auria, Gianna Palmieri, Marta Gogliettino, Bruna Agrillo, Rosarita Tatè, Principia Dardano, Luigi Nicolais, Marco Balestrieri

**Affiliations:** 1 Department of Pharmacy, University of Naples Federico II, Via D. Montesano 49, 80131 Naples, Italy; lucia.falcigno@unina.it (L.F.); gabriella.dauria@unina.it (G.D.); 2 Institute of Biosciences and BioResources, National Research Council (IBBR-CNR), Via Pietro Castellino 111, 80131 Naples, Italy; bruna.agrillo@ibbr.cnr.it (B.A.); marco.balestrieri@ibbr.cnr.it (M.B.); 3 Materias Srl, Corso N. Protopisani 70, 80146 Naples, Italy; nicolais@unina.it; 4 Department of Biology, University of Naples Federico II di Monte Sant’Angelo, Via Cintia 21, 80126 Naples, Italy; 5 Institute of Genetics and Biophysics, National Research Council (IGB-CNR), Via Pietro Castellino 111, 80131 Naples, Italy; rosarita.tate@igb.cnr.it; 6 Institute of Applied Sciences & Intelligent Systems, National Research Council (ISASI-CNR), Via Pietro Castellino 111, 80131 Naples, Italy; principia.dardano@na.isasi.cnr.it

**Keywords:** AMP, NMR, stereomicroscopy, CD, atomic force microscopy (AFM), fluorescence, self-assembling, conformation distribution

## Abstract

Antimicrobial peptides (AMPs) represent a skilled class of new antibiotics, due to their broad range of activity, rapid killing, and low bacterial resistance. Many efforts have been made to discover AMPs with improved performances, i.e., high antimicrobial activity, low cytotoxicity against human cells, stability against proteolytic degradation, and low costs of production. In the design of new AMPs, several physicochemical features, such as hydrophobicity, net positive charge, propensity to assume amphipathic conformation, and self-assembling properties, must be considered. Starting from the sequence of the dodecapeptide 1018-K6, we designed a new 10-aminoacid peptide, namely RiLK1, which is highly effective against both fungi and Gram-positive and -negative bacteria at low micromolar concentrations without causing human cell cytotoxicity. In order to find the structural reasons explaining the improved performance of RiLK1 versus 1018-K6, a comparative analysis of the two peptides was carried out with a combination of CD, NMR, and fluorescence spectroscopies, while their self-assembling properties were analyzed by optical and atomic force microscopies. Interestingly, the different spectroscopic and microscopic profiles exhibited by the two peptides, including the propensity of RiLK1 to adopt helix arrangements in contrast to 1018-K6, could explain the improved bactericidal, antifungal, and anti-biofilm activities shown by the new peptide against a panel of food pathogens.

## 1. Introduction

In recent decades, growing interest in antimicrobial peptides (AMPs) for their applications as bactericidal agents in different fields has been observed. Firstly, the increased bacterial resistance due to the uncontrolled use of antibiotics has raised significant concerns in medicine, encouraging research into novel therapeutics [1,2,3]. In this scenario, AMPs are considered a promising new class of antibiotics [4]. Moreover, another relevant application of AMPs is that in the food industry as antimicrobials [5,6,7,8,9,10,11], bio-preservatives [12], and anti-biofilm agents [3,13,14]. Indeed, AMPs, also known as host defense peptides (HDPs), are an important group of natural substances widely distributed in nature, offering a broad spectrum of activity against Gram-positive and Gram-negative bacteria, viruses, fungi, and parasites [2]. A wide variety of organisms, ranging from prokaryotes to humans, produce AMPs as a part of their first line of defense [15]. In addition, AMPs isolated from natural sources as well as their synthetic variants have revealed their broad-spectrum antimicrobial activity [16,17,18]. AMPs are small, predominantly cationic and amphipathic polypeptides, with different compositions, molecular masses and secondary structures [2,13]. However, AMPs exhibit several structural characteristics which are essential for their activity [17]. First, to interact with and penetrate bacterial membranes, AMPs must show a right balance of positive charge and hydrophobicity [19,20]. Indeed, the net positive charge enables electrostatic attractions between AMPs and the negatively charged microbial membranes, whilst hampering the interactions with the neutrally charged mammalian cell membranes [15,21,22,23]. At the same time, the hydrophobicity of AMPs allows them to penetrate cells, inducing membrane lysis [24,25]. Due to the complexity of peptide–membrane interaction, the hydrophilicity/hydrophobicity properties must be finely balanced to optimize the activity and selectivity of AMPs avoiding cytotoxicity [20]. In this regard, the conformational features of AMPs play a key role [4,26]. Peptides show different conformations and aggregation states in free or in membrane-bound form [4,27]. Such items deeply influence the efficacy and selectivity of AMPs. Specifically, the self-assembling properties of AMPs have received increasing attention in the rational design and engineering of smart AMPs [28,29,30,31,32].

Recently, Palmieri et al. [33] projected a peptide, namely 1018-K6, designed on the basis of the dodecapeptide IDR-1018, a natural derivative of a bovine HDP bactenecin, which belongs to the cathelicidins family [34]. Cathelicidins are small, cationic, antimicrobial peptides found in humans and other species, including farm animals. The only cathelicidin in humans is the cationic protein of 18 kDa (hCAP18), which is expressed in neutrophils, monocytes, and epithelial cells [35], stored as an inactive precursor and processed to generate the active peptide LL-37 [35]. These proteolytically activated peptides are part of the innate immune system in many vertebrates and show a broad spectrum of antimicrobial activity against bacteria, enveloped viruses and fungi. Apart from exerting direct antimicrobial effects, cathelicidins can also trigger specific defense responses in the host [35].

Although native IDR-1018 shows a broad spectrum of antimicrobial and anti-biofilm activity [36,37,38,39], the analogue 1018-K6 revealed a stronger efficiency than IDR-1018 in eradicating and inhibiting biofilm growth as well as in killing the planktonic cells of *Listeria* strains isolated from food-products and food-processing environments [33,40]. On these grounds, to further improve the structural features and the antibacterial performances of 1018-K6 peptide, some modifications were planned taking into account (i) to shorten the peptide length in order to reduce the costs of production and to improve the stability and safety; (ii) to take the proper content of basic and hydrophobic residues; and (iii) to ensure the formation of an amphipathic helix. The best obtained analogue, a decapeptide named RiLK1 (RLKWVRIWRR-NH2), has been synthesized and characterized [41]. RiLK1 was highly effective against both fungi and Gram-positive and -negative bacteria, with no evidence of cytotoxicity on human keratinocytes and fibroblasts.

In this paper, we reported the structural characterization of RiLK1 and 1018-K6 peptides in water and in SDS micellar solutions carried out by a combination of NMR, CD and fluorescence spectroscopic techniques. As generally observed for AMPs, conformational changes are observed moving from pure water to micellar environments as well as for our peptides, even though they show different structural behavior. In order to investigate their propensity for self-assembling, RiLK1 and 1018-K6 were analyzed by optical and atomic force microscopies. Our results point out a conformational propensity of RiLK1 to self-assemble in regular structures more than 1018-K6. This structural finding could explain the excellent bactericidal, antifungal and anti-biofilm activities exhibited by RiLK1 in comparison to 1018-K6 against a panel of food-pathogens [41].

## 2. Results

### 2.1. Molecular Design of RiLK1 Peptide

AMPs are known to be highly variable in terms of size and sequence and can be classified according to their secondary structure (α-helix, β-sheet, etc.) [15,42]. These peptides are rich in basic amino acids (Lys, Arg) that confer an overall net charge ranging from +2 to +9, and they possess approximately 50% hydrophobic residues, which may favor an amphipathic conformation upon interaction with membranes [4].

Specifically, the amphipathic topology is considered a key aspect for the AMP’s mechanism of action. Indeed, in this state, the positively charged surface of AMP guarantees the contact with the negatively charged surface of the bacterium, while its hydrophobic surface can get in touch with the internal portions of the membrane, destabilizing and finally breaking it [43].

When designing a new AMP, Arg and Trp residues are preferred among positively charged and hydrophobic amino acids, respectively, for their properties of interaction with the bacterial membranes [18]. Indeed, the Arg residue endows the peptides with cationic charges, and it has a higher pKa and multi-dentate hydrogen bonding properties that favor the interaction with membranes compared to Lys [43,44,45,46]. As regards the Trp residue, the flat rigid shape and the aromaticity (i.e., its π electron structure and associated electrical quadrupole moment) of side-chain promote its penetration and residing in the interface environment [18,46].

Based on all these evidences, the peptide RiLK1 (RLKWVRIWRR-NH_2_) was de novo designed starting from the 1018-K6 sequence (VRLIVKVRIWRR-NH_2_) [33]. The new decapeptide analogue holds the last six C-terminal residues of the parent dodecapeptide 1018-K6, while in the N-terminal portion, it is shortened by three residues and modified by the insertion of a Trp residue [41]. It is worth noting that the molecular design has successfully produced a more performing AMP, as RiLK1 exhibits greatly improved antimicrobial efficiency compared to the parent 1018-K6 [41].

### 2.2. Solid-State Analysis of RiLK1 and 1018-K6 by AFM and Optical Microscopy

The self-assembling properties of RiLK1 and 1018-K6 peptides were evaluated by atomic force microscopy (AFM) and stereomicroscopy.

The AFM study was performed on washed and dried samples (see Materials and Methods section for the detailed sample preparation) obtained from peptides solved in PBS and in water in order to compare the aggregation tendency of both molecules in the presence or absence of salts. Sample preparation provides the gentle and extensive rinsing of the surface with deionized water (5 mL per sample), which ensures the complete removal of salt crystals and the quote of peptide not bound on mica surface.

**Figure 1:**
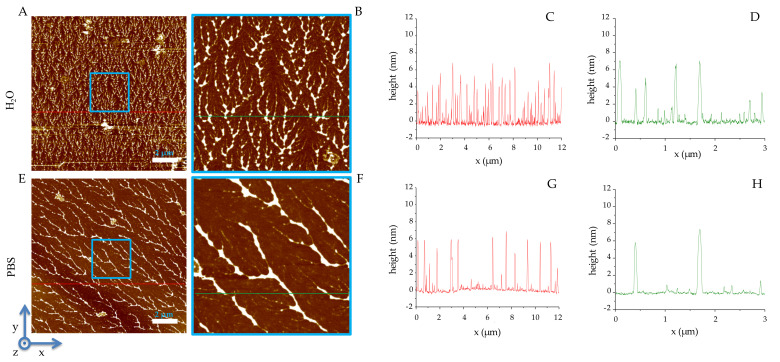
AFM images of RiLK1. (**A**) 12 μm × 12 μm field image of dried water solution of RiLK1, the scale bar is 2 μm, the blue square locates the 3 μm × 3 μm field reported in image (**B**); heights referred to by the red line in (**A**) are reported in the graph line in (**C**) and the heights referred to by the green line in (**B**) are reported in the graph line in (**D**); (**E**) 12 μm × 12 μm field image of dried PBS solution of RiLK1, the scale bar is 2 μm, the blue square locates the 3 μm × 3 μm field reported in image (**F**); heights referred to by the red line in (**E**) are reported in the graph line in (**G**) and heights referred to by the green line in (**F**) are reported in the graph line in (**H**).

In both environments, RiLK1 self-aggregates in linearly ordered structures (Figure 1), which are more tightly distributed on the mica surface when obtained from water than from PBS (see Figure 1A,B vs. E,F), although the heights of the aggregates are identical (≈6 nm), as shown in the profiles reported in Figure 1C,D,G,H.

**Figure 2:**
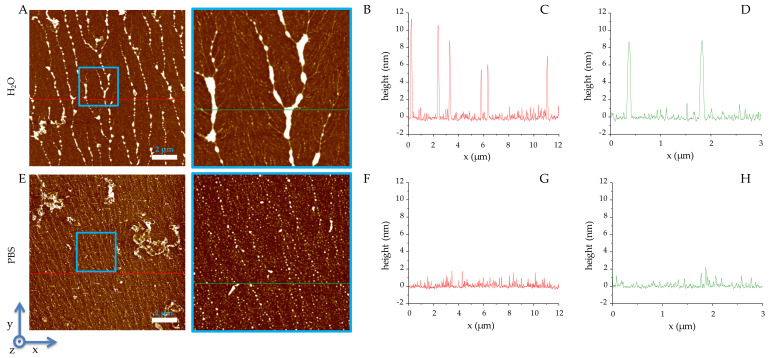
AFM images of 1018-K6. (**A**) 12 μm × 12 μm field image of dried water solution of RiLK1, the scale bar is 2 μm, the blue square locates the 3 μm × 3 μm field reported in image (**B**); heights referred to by the red line in (**A**) are reported in graph line in (**C**) and heights referred to by the green line in (B) are reported in graph line in (**D**); (**E**) 12 μm × 12 μm field image of dried PBS solution of RiLK1, the scale bar is 2 μm, the blue square locates the 3 μm × 3 μm field reported in image (**F**); heights referred to by the red line in (**E**) are reported in graph line in (**G**) and heights referred to by the green line in (**F**) are reported in graph line in (**H**).

On the other hand, 1018-K6 self-assembles in linearly ordered structures (Figure 2) from water with a mean height of approximately 8 nm (Figure 2C,D), whilst in PBS a diffuse thin layer of molecules with a height of about 1 nm is observed (Figure 2G,H).

The propensity of peptides to self-assembling was also investigated by stereomicroscopy. This analysis, performed on dried samples obtained from RiLK1 and 1018-K6 solutions at different concentrations (40, 80, 160, 240 μM) in PBS and in phosphate buffer (PB) indicates that the two peptides self-assemble in structures of different shapes, where, in the presence of salt, 1018-K6 aggregates in flat layers, while RiLK1 aggregates by forming tri-dimensional structures (Supplementary Figures S1 and S2).

### 2.3. Structural Analysis of RiLK1 and 1018-K6 by CD and Fluorescence Spectroscopies

In order to study the structural features of RiLK1 and 1018-K6 and their aptitude to interact with membranes, an extensive spectroscopic investigation was performed by CD and fluorescence spectroscopies in water and in SDS micelles, a widely used model system to study interactions between AMPs and cell membranes [9,10,33,40,41,47,48,49]. It is worth noting that this surfactant may be suitable for all the spectroscopic techniques performed in this work and even more for NMR analysis in solution. In addition, our results can easily be compared with the extensive literature on AMPs, which reports the functional data combined with studies in SDS micellar solutions [9,10,33,40,41,47,48,49].

The behavior of the two peptides in response to various factors such as SDS concentration, SDS/peptide ratios, pH values, and incubation time was examined.

#### 2.3.1. Effect of SDS Concentration on Peptide Structure

**Figure 3:**
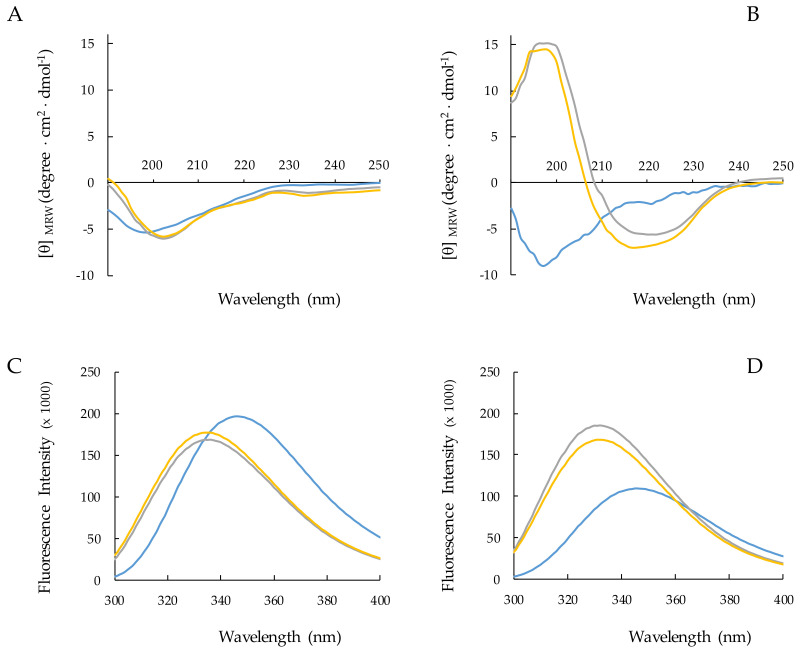
CD and fluorescence spectra of RiLK1 and 1018-K6. RiLK1 (**A**,**C**) and 1018-K6 (**B**,**D**) were analyzed at increasing SDS concentration. Spectra were recorded at peptide concentration of 50 μM, pH 4.0 and 25 °C in the absence of SDS (blue lines) and in the presence of SDS 50 mM (gray lines) or 150 mM (yellow lines).

The CD spectra of the two peptides in water and in the presence of different concentrations of SDS are shown in Figure 3A,B. Spectral data were analyzed using the K2D software to estimate the contributions to the spectrum of the secondary structure elements α-helix, β-sheet, and coil [50].

RiLK1 (Figure 3A) and 1018-K6 (Figure 3B) adopt in water a random coil conformation as indicated by the presence in the CD spectra of a negative band at 198 nm. The addition of SDS to the peptide solutions induces spectral variations that are different for the two peptides. Concerning RiLK1, the minimum shifts to ~204 nm and a shallower negative peak appears at ~235 nm upon the addition of 50 or 150 mM SDS. These variations reveal that SDS induces changes in the peptide conformational distribution by somehow stabilizing the ordered structures ɑ-helix (27%) and β-sheet (37%) with respect to the random coil structure (40%) (Figure 3A, Supplementary Table S1). A different behavior was observed for 1018-K6 which responds to the presence of SDS stabilizing at 100% in β-structure. It is worth noting that the use of prediction algorithms to estimate the secondary structure composition from CD spectra often fails to provide adequate results on ɑ/β-mixed or β-structure peptides. However, the CD conformational distributions observed for the two peptides in the presence of SDS provided structural clues that were confirmed by the NMR analyses.

To complement the CD data, the same analyses were performed by fluorescence, taking advantage of the presence of tryptophan residues in both peptide sequences. In water, the maximal fluorescence emission (λmax) for both peptides was observed at ~350 nm, a value that is typical for Trp indole group fully exposed to hydrophilic environments. The addition of SDS causes a blue shift of λmax from 350 to 335 nm in the fluorescence spectra of both peptides (Figure 3C,D). This effect is observed when the tryptophan side chain shifts from a hydrophilic to a less polar and/or less dynamic surrounding solvent. However, the two peptides display changes in fluorescence intensity of opposite signs with the transfer from the aqueous to the SDS environment. Indeed, while 1018-K6 shows an increase in the fluorescence intensity consistent with a decreased flexibility of its Trp residue, a quench in the emission band was evidenced for RiLK1, probably suggesting that one or both Trp residues are involved in hydrophobic interactions in the micellar solutions.

Taken together, the CD and fluorescence analyses suggest that both peptides are able to interact with SDS micelles, even though stabilizing in different type conformations: i.e., α-helix for RiLK1, β-sheet for 1018-K6.

#### 2.3.2. Peptide–SDS Interaction: The Peptide Concentration Effect

The dependence of peptide–micelle interaction on the peptide concentration was followed by CD and fluorescence. Spectra were acquired at 12.5, 25 and 50 µM concentration of each peptide in 50 mM SDS, at pH 4.0 (Supplementary Figures S3 and S4).

Regarding the CD behavior of RiLK1, the spectral analysis obtained by K2D shows an increase in the α-helix component at the expense of the β-sheet structure, as the concentration of the peptide increases (Supplementary Table S2A). On the opposite, 1018-K6 predominantly folded in β-sheet conformation upon titration with increasing peptide concentrations (Supplementary Table S2B). In the fluorescence spectra, the extent of the λmax blue shift is independent of the concentration of the peptide, whether it is RiLK1 or 1018 K6 (Supplementary Figures S3 and S4). However, the fluorescence intensities change with an opposite trend for the two peptides. As the concentration of 1018-K6 increases, the emission intensity rises while for RiLK1, the increment in the peptide concentration causes a decrease in the fluorescence band (quenching).

In summary, the CD investigation shows that in the presence of SDS, and at increasing concentrations, both peptides stabilize in more ordered, albeit different, structures: α-helix for RiLK1 and β-sheet for 1018-K6. Furthermore, the fluorescence data suggest that RiLK1 interacts with the SDS micelle better than 1018-K6.

#### 2.3.3. Peptide–SDS Interaction: Time Effect

**Figure 4:**
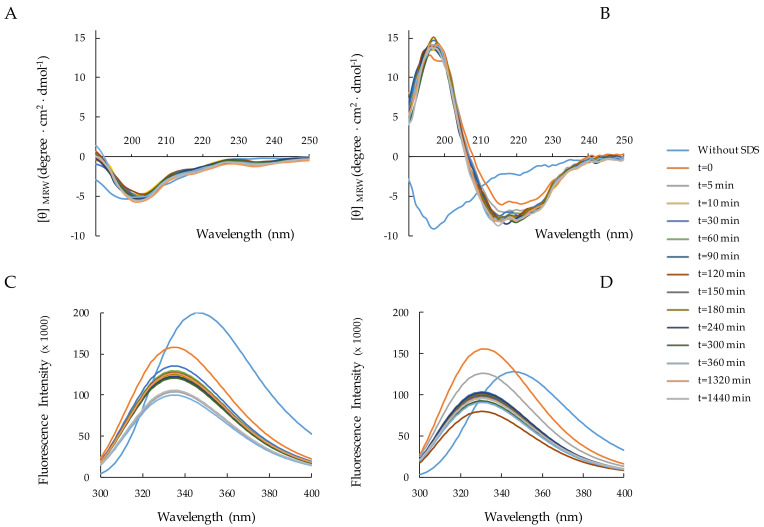
CD and fluorescence spectra of RiLK1 and 1018-K6. RiLK1 (**A**,**C**) and 1018-K6 (**B**,**D**) were analyzed as a function of time. Spectra were recorded at a peptide concentration of 50 μM in 150 mM SDS, at pH 4.0 and at 25 °C after up to 24 h incubations. Spectra of peptides 50 μM in water, without SDS (blue lines), were also reported.

We studied the peptide folding kinetics of RiLK1 and 1018-K6 in the presence of 150 mM SDS during the 24 h incubation. The CD spectra (Figure 4A,B) evidence that each peptide retains its own conformational distribution (Supplementary Table S3A,B) during the time. Conversely, the fluorescence spectra (Figure 4C,D) change significantly over time, showing a decrease in the signal intensity for both peptides. This quenching was probably due to self-assembling or the strengthening of interactions with SDS micelles, or both.

#### 2.3.4. Peptide–SDS Interaction: pH and Temperature Effects

Finally, as the antibacterial activity of many AMPs is attenuated by several physico-chemical parameters, the conformational stability of RiLK1 and 1018-K6 at different pH and temperatures for 48 h in SDS micellar solutions was investigated (Supplementary Figures S5 and S6). As far as 1018-K6 is concerned, the dichroic spectra clearly manifest a stabilization of β-sheet conformation at acidic and neutral pH (Supplementary Figure S5A,B and Supplementary Table S4A), with fractional helicity increasing from ∼9% to 60% over experimental time span—but only at basic pH values (Supplementary Figure S5C and Supplementary Table S4A). On the contrary, RiLK1 evidences a dynamic equilibrium among different folds, at the different pH levels over 48 h incubation (Supplementary Figure S6 and Supplementary Table S4B). As for the fluorescence data, the maximal fluorescence intensity and the fluorescence emission maximum in the presence of micelles are independent of the pH values both for RiLK1 and 1018-K6, indicating that emitting tryptophan residues are located in similar environments, except for RiLK1 at acidic pH values that produced a decrease in fluorescence quantum yields (Supplementary Figures S5 and S6).

A high structural and folding stability was observed, also subjecting RiLK1 and 1018-K6 to thermal treatments that did not induce any significant changes in the CD and fluorescence spectra at all the temperatures investigated, although 1018-K6 seemed to be less thermally stable at 90 °C with respect to RiLK1 (Supplementary Figures S7 and S8 and Supplementary Table S5).

### 2.4. Structural Analysis of RiLK1 and 1018-K6 by NMR

#### 2.4.1. NMR Analysis in Water

One-dimensional NMR spectra are in the Supplementary Material (Supplementary Figure S9).

**Figure 5:**
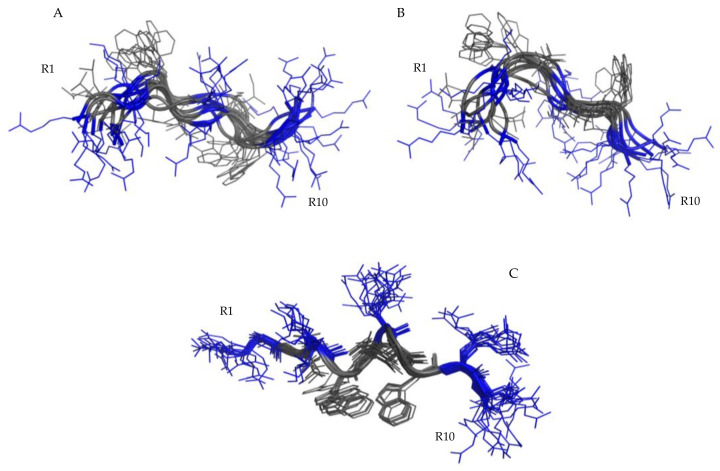
NMR structures of RiLK1. Superimposition of structures belonging to the first two Chimera clusters representative of RiLK1 conformation in water: (**A**) cluster 0, 9 models; (**B**) cluster 1, 8 models; (**C**) superimposition of 20 structures belonging to the main Chimera cluster representative of RiLK1 conformation in SDS. Cartoon representation with hydrophobic side-chains as lines colored in gray and positive charged (**R**,**K**) in blue.

**Figure 6:**
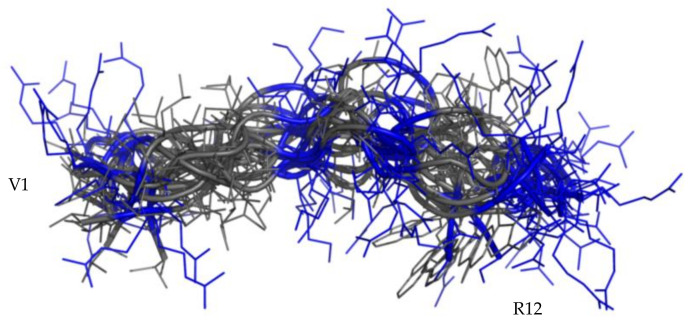
NMR structures of 1018-K6. Superimposition of 17 structures belonging to the main Chimera cluster representative of 1018-K6 conformation in water. Cartoon representation with hydrophobic side-chains as lines colored in gray and positive charged (R, K) in blue.

NMR data, such as the deviations of the αCH proton chemical shift (Supplementary Figure S10) from random coil values [51] and NOEs, collected for RiLK1 and 1018-K6 in H2O/D2O 90/10 at pH 4, indicate random coil conformations of both peptides. This structural diagnosis was confirmed by the molecular models computed by the CYANA program [52] using NOE-derived distances as the upper limit (upl) of interproton distances (for details see Table 1). The best 40 CYANA structures in terms of agreement with experimental data, i.e., with the lowest target function (TF) values (Table 1), were clustered by the CHIMERA program [53]. The structures contained in the first most-populated clusters were chosen as representative of the conformational space accessible to the peptides and reveal a huge conformational flexibility of RiLK1 (Figure 5A,B) and 1018-K6 (Figure 6) in pure water.

#### 2.4.2. NMR Analysis of RiLK1 in SDS Micelles

The conformational NMR analysis was performed in a micellar environment of SDS/water mixture (150 mM SDS, pH 4.4) only for RiLK1, whilst 1018-K6 at millimolar concentration was found insoluble in this *medium*. In the micellar environment, RiLK1 adopts a relatively ordered structure. The negative αCH deviations from random coil values < −0.1 ppm [48] (Supplementary Figure S10) point to a helical conformation in middle/C-terminal regions of the peptide. This structural diagnosis is consistent with the NOE pattern (Figure S11). Indeed, the presence of NOE effects, such as NHi-NHi+1, together with long range αi-Ni+2, αi-Ni+3 and αi-βi+3 contacts, indicate the occurrence of a helical structure in the central region of the peptide. Structural calculations were carried out by CYANA [52] using 156 NOE-derived distances as the upper limit (upl) of inter-proton distances (130 intra-residues, 22 sequential, 4 long range), as reported in Table 1. The 40 lowest TF CYANA structures were clustered by the CHIMERA program [53]. The 20 structures contained in the first and most-populated cluster (Figure 5C) were chosen as representative of the conformational space accessible to the peptide. Their superimposition shows a well-defined structure in helix arrangement in K3-W8 segment (RMSD value on the backbone atoms of 0.26 Å) which places positively charged and hydrophobic side-chains on opposite sides. It is worth noticing that, in this arrangement, all the charged side-chains (R1, K3, R6, R9, R10) are iso-oriented and well prone to interact with the negatively charged surface of SDS micelles. Moreover, the two Trp side chains are located at a proper distance for mutual stacking, so contributing to the helix stabilization.

The atomic coordinates are deposited in the Protein Data Bank (PDB code 70B2).

## 3. Discussion

The 12-mer 1018-K6 and the 10-mer RiLk1 are two peptides designed to function as antimicrobials, with the second one, rationally projected using the sequence of the first one as a template.

Previously, results demonstrated that RiLK1 exhibited a stronger killing efficiency than the 1018-K6 peptide against *Listeria monocytogenes* and *Salmonella typhimurium*, as well as against fungal pathogens [41].

The structural reasons that make RiLK1 a more efficient antimicrobial than 1018-K6 have been explored herein by comparing the two peptides, both with respect to their ability to self-aggregate, and for their conformational and interaction profiles with a bacterial membrane mimic.

The AFM analysis demonstrates that both RiLK1 and 1018-K6 are able to form ordered structures when their solutions are deposited on mica plates. Nevertheless, while 1018-K6 only aggregates at a low salt content, RiLK1 is organized into ordered aggregates both in water and in the presence of PBS, proving to be less sensitive to environmental conditions.

In water and at pH 4, both peptides are disordered, while they interact differently with SDS micelles. As previously reported, the addition of SDS below the critical micellar concentration produces in both peptides the appearance of a beta conformation in equilibrium with a random structure [40,41]. However, in SDS micelles, 1018-K6 stabilizes almost 100% in beta-sheet structure, while RiLK1 shows the co-existence of multiple α-helical and/or β-sheet-like subpopulations, which are in fast equilibrium with unordered states and whose formation is favored by the highly dynamic nature in the solution of small peptides like RiLK1. Therefore, the interaction with various proportions of SDS micelles could occur at several stages and a process of a simple two-state equilibrium could not sufficiently describe all the observed structural changes (Supplementary Table S1).

Interestingly, keeping the SDS concentration constant (50 mM), the increase in the peptide concentration causes the stabilization of the beta structure in 1018-K6 in contrast to RiLK1, showing a more complex conformational distribution (Supplementary Table S2).

The effects of SDS on the peptide structure can derive from the variation of their chemical–physical properties, or from self-assembling processes, or be the result of a peptide–micelle interaction. In the latter case, the fact that RiLK1 in the presence of SDS shows the quenching of the fluorescence emission while 1018-K6 does not, which suggests that the interaction of RiLK1 with the SDS micelles is tighter than that of 1018-K6. This hypothesis is in line with the observation that RiLK1 is soluble at millimolar concentrations in the presence of 150 mM SDS, while 1018-K6 is not. Since these conditions are those suitable for NMR measurements, only RiLK1 could be characterized in the presence of SDS micelles with this technique.

The NMR analysis confirms the CD structural diagnosis. In pure water, both peptides adopt iso-energetic disordered structures in fast inter-conversion on the NMR time scale (Figure 5 and Figure 6), showing a random orientation of both charged and hydrophobic side chains. Notwithstanding, we found that approximately 25% of the structures calculated in water for RiLK1 exhibit a tendency to an amphiphilic arrangement (Figure 5B), which is definitively stabilized in the presence of SDS micelles (Figure 5C). In RiLK1, the Trp residue inserted in position 4 creates a hydrophobic cluster with the other Trp residue (W8). Such a modification affects the ability of RiLK1 to adopt amphipathic structures and improves the peptide binding to the micelles. Indeed, the structural model obtained by NMR for RiLK1 in SDS, shows a helix structure in the K3-W8 segment. The amphipathic helix is stabilized on one face by the presence of a Trp cluster and on the other by the interaction between the positively charged and the negatively charged SDS micelles.

The amphiphilic arrangement is functional to the formation of ordered self-aggregates. Indeed, the most investigated mechanisms of antimicrobial action foresee that peptides can act not only as single entities but also in self-assembled forms to form channels that pierce the membrane (pore mechanism) or to form layers that cover the membrane destabilizing it (a carpet-like mechanism) [25,54].

Although RiLK1 and 1018-K6 share 75% of their sequence identity, their antibacterial activity, their behavior in terms of self-assembling, their conformational propensities and so their interaction skills with micelles, are different. The presence of SDS stabilizes the two peptides in ordered structures. The effect on 1018-K6 is to tighten its conformational distribution to the 100% of the beta form. Therefore, the micelles favor a beta-sheet self-assembling for 1018-K6 but further studies will be needed to establish its antibacterial mechanism of action.

On the contrary, the effect of SDS on RiLK1 is to widen the conformational distribution. The peptide is shorter and less prone to order than 1018-K6. Therefore, RiLK1 may have a wider spectrum of mechanisms of action, depending on the specific characteristics of the membrane with which it comes into contact. For all these features, RiLK1 represents a promising candidate for a new class of peptide-based antibiotics.

## 4. Materials and Methods

### 4.1. Optical and Atomic Force Microscopy

RiLK1 and 1018-K6 were re-suspended in Dulbecco’s phosphate buffered saline 1x (DPBS, Corning, Glendale, AZ, USA) or in phosphate buffer (PB) at different concentrations (40-80-160-240 μM), in a 48 multi-well-dish (200 μL/well), dried O/N at 60 °C and finally observed by stereomicroscope Leica MZ16-FA (Wetzlar, Germany) at different magnification. Images were recorded digitally with a Levenhuk M1000 PLUS camera (Levenhuk srl, Giulianova (TE), Italy). Sample preparation for the AFM study follows the procedure reported [55,56]. Briefly, muscovite mica is used as a superhydrophilic and atomically flat areas substrate with a wide presence of highly mobile K+ ions after cleavage. The deposition of peptides was realized by casting 3 μL aliquots for each imaged sample (RiLK1 and 1018-K6 in PBS or in water) onto a freshly cleaved muscovite mica. Each aliquot was left on the mica for 2 min to bind the K+ charged mica surface and the negative domains of RiLK1 and 1018-K6 compound, and then extensively rinsed with deionized water (5 mL per sample) in order to completely remove salt crystals and the quote of peptide not bound to the mica surface. Therefore, the peptide samples were dried by evaporation at room temperature under a ventilated fume hood [57]. For AFM investigation, the concentrations of both peptides were 80 μM in order to obtain the clearest images under dried conditions. The images were obtained by using A XE-100 AFM (Park Systems, Suwon, South Corea). Surface imaging was recorded in non-contact mode using silicon/aluminum coated cantilevers (SSS-NCHR 10M; Park Systems) 125 μm-long, with a resonance frequency of 204 to 397 kHz, a nominal force constant of 42 N/m and a typical tip radius 2 nm (<5 nm max). The scan frequency was typically 0.5 Hz per line for 1064 × 1064-pixel image. Usually, the AFM images are flattened in order to remove the background slope and the contrast and brightness are adjusted. For each sample and for each concentration, the analyses were recorded three times.

### 4.2. CD Spectroscopy

Circular dichroism (CD) analysis was performed by the Jasco J-810 spectropolarimeter. The samples were loaded into a quartz cuvette of 0.1 cm path length (Hellma Italia srl, Milano, Italy) and the spectra were recorded in the 190–250 nm range at a scan speed of 20 nm/min, by averaging 5 scans and in the presence or absence of SDS (Sigma Aldrich, Milano, Italy). The effect of pH on the secondary structure of RiLK1 and 1018-K6 was evaluated by dissolving the samples at concentration of 50 **¦**μM in water at pH 2.0, pH 7.0 and pH 12.0. Then, SDS (150 mM final concentration) was added to each sample, which was incubated for further 48 h at 25 °C and analyzed by CD spectroscopy. The folding kinetic measurements of the peptides were carried out after the addition of SDS (150 mM) to each sample (50 **¦**μM in water pH 4.0) up to 24 h incubation. CD experiments were also performed in water at pH 4.0 as function of peptide concentration or SDS at a peptide concentration of 50 **¦**μM. For the thermal stability, the peptides were prepared to a final concentration of 50 μM in water at pH 4.0 in the presence of 150 mM SDS, and then, they were incubated at 4, 37 and 90 °C up to 48 h before acquiring the CD spectra. In all analyses, the percentage of secondary structure was estimated by the DICHROWEB site [58,59,60], using the algorithm K2D [50]. The mean residue ellipticity ([θ], deg. Cm^2^ dmol^−1^) was obtained by the equation [θ] = 100 θ/cnl, where θ is the ellipticity (mdeg), c is the peptide concentration (mM), n is the number of residues, and l is the path length (cm). For each sample, the background (buffer) was subtracted automatically from the signal.

### 4.3. Fluorescence Spectroscopy

Trp fluorescence emission spectra were recorded at 25 °C on a Shimadzu RF-6000 spectrofluorometer (Kyoto, Japan) with both excitation and emission slit widths set at 5 nm. The intrinsic tryptophan was excited at a wavelength of 280 nm and the emission was monitored between 300 and 400 nm. The folding kinetic experiments of RiLK1 and 1018-K6 were carried out after the addition of SDS (150 mM) to each sample (50 μM concentration in water pH 4.0) up to 24 h incubation. Fluorescence measurements were also performed in water pH 4.0 as a function of peptide concentration or SDS at a peptide concentration of 50 μM. The effect of pH on peptide folding was evaluated by dissolving the peptides at a final concentration of 50 μM in water at pH 2.0, 7.0 or 12.0. Then, the SDS (150 mM final concentration) was added to each sample, which was incubated up to 48 h at 25 °C and analyzed by fluorescence spectroscopy. For the thermal stability, the peptides were prepared to a final concentration of 50 μM in water at pH 4.0 in the presence of 150 mM SDS and then they were incubated at 4, 37 and 90 °C up to 48 h.

### 4.4. NMR Spectroscopy

The solution structures for NMR analysis of RiLK1 and 1018-K6 peptides were determined under different conditions: pure water (H_2_O/D_2_O 90/10 v/v) pH 4 and in micellar SDS (H_2_O/SDS-d25 150 mM, pH 4.4). Deuterated water (98% isotopic purity) and SDS-d25 (98% isotopic purity) were purchased by Sigma Aldrich, Milano, Italy. NMR spectra were recorded on a Varian Unity Inova 700MHz Spectrometer located at the Department of Pharmacy—University of Naples “Federico II”. The 2D 1H-NMR, TOCSY (mixing time 70 ms) and NOESY (mixing time 300 ms) were recorded at 298 K. The water resonance was suppressed by the use of gradients. Chemical shifts were referred to internal sodium 3-(trimethylsilyl) propionate 2,2,3,3-d4 (TSP, Sigma Aldrich, Milano, Italy). NMR spectra were analyzed by using CARA program (http://cara.nmr.ch/doku.php/home, accessed on 10 March 2021). Proton resonances were sequentially assigned by following the Wuthrich standard method [61].

NMR data were deposited in the Biological Magnetic Resonance Bank (BMRB entry 50902).

### 4.5. Computational Methods

NOE intensities, evaluated by the integration of cross-peaks in the 300 ms NOESY spectra, were converted into inter-proton distances by the use of the CALIBA program [62]. Geminal protons were chosen as the reference with a distance of 2.2 Å.

Structure calculations started from 100 randomized conformers and used the standard CYANA simulated annealing schedule with 20000 torsion angle dynamics steps per conformer [52]. Three dimensional structures were obtained by using inter-proton distances evaluated from NOEs as upper limits (Table 1). All the conformers showed fairly good agreement with the experimental constraints showing no violations. The best 40 CYANA structures out of 100 calculated structures, in terms of agreement with experimental data, i.e., with the lowest target function (TF) values (Table 1), were clustered by the CHIMERA program [53]. The molecular structures were visually analyzed by PyMOL software Molecular Graphics System, Version 2.0 Schrödinger, LLC.

## 5. Patents

Application No: PCT/IB2021/052519 Publication Date: 26/03/2021. Antimicrobial Peptides. BALESTRIERI MARCO, PALMIERI GIANNA, NICOLAIS LUIGI.

## Figures and Tables

**Table 1:** CYANA structural statistic of RiLK1 and 1018-K6 in different media.

Structural Parameters	RiLK1 in SDS ^a^	RiLK1 in Water	1018-K6 in Water
distance restraints	156	120	123
intra-residue	130	106	109
sequential (|i − j| = 1)	22	14	14
Medium range (1< |i − j| ≤ 4)	4	0	0
Violation Statistics (40 structures)			
CYANA TF (Å^2^)	0.26 ± 0.02	2.1 × 10^−2^ ± 2.1 × 10^−2^	1.13 × 10^−2^ ± 9.9 × 10^−3^
Residual Distance Constraint Violations (Å)			
number > 0.2 Å	0	0	0
mean global backbone RMSD	0.81 ± 0.36 Å	2.15 ± 0.41 Å	2.60 ± 0.42 Å
mean global heavy atom RMSD	2.04 ± 0.45 Å	4.36 ± 0.58 Å	4.53 ± 0.51 Å
mean global heavy atom RMSD	2.04 ± 0.45 Å	4.36 ± 0.58 Å	4.53 ± 0.51 Å

^a^ SDS 150 mM, pH 4.

## Data Availability

The NMR structures of RiLK1 in SDS are available in the Protein Data Bank: PDB code 70B2. Proton chemical shifts of RiLK1 in SDS are available in Biological Magnetic Resonance Bank: BMRB entry 50902.

## Supplementary Materials

The following are available online at https://www.mdpi.com/article/10.3390/ijms221810011/s1, Figure S1: Stereomicroscopic analysis of RILK1 and 1018-K6 in PBS; Figure S2: Stereomicroscopic analysis of RILK1 and 1018-K6 in PB; Figure S3: CD (left) and fluorescence (right) spectra of RiLK1 at increasing peptide concentration; Figure S4: CD (left) and fluorescence (right) spectra of 1018-K6 at increasing peptide concentration; Figure S5: Effect of pH on the folding and secondary structure of 1018-K6 monitored by spectroscopic techniques; Figure S6: Effect of pH on the folding and secondary structure of RiLK1 monitored by spectroscopic techniques; Figure S7: Thermostability of 1018-K6 monitored by spectroscopic techniques; Figure S8: Thermostability of RiLK1 monitored by spectroscopic techniques; Figure S9: 1H NMR spectra of RiLK1 1.0 mM; Figure S10: Chemical shift deviations (ppm) from random coil values of aCH protons; Figure S11: Most relevant NOE effects measured for RiLK1 peptide in SDS; Table S1: Secondary structure contents of (A) RiLK1 and (B) 1018-K6 at 50 μM concentration in water pH 4 in the presence of different concentrations of SDS determined by the Dichroweb server (K2D); Table S2: Secondary structure contents of (A) RiLK1 and (B) 1018-K6 at different concentrations in water pH 4.0 in the presence of 50 μM SDS determined by the Dichroweb server (K2D); Table S3: Secondary structure contents of (A) 1018-K6 and (B) RiLK1 50 μM concentration in water pH 4 in the presence of 150 mM SDS at different times determined by the Dichroweb server (K2D); Table S4: Secondary structure contents of (A) 1018-K6 and (B) RiLK1 at 50 μM concentration in water at different pHs in the presence of 150 mM SDS determined by the Dichroweb server (K2D); Table S5: Secondary structure contents of (A) 1018-K6 and (B) RiLK1 at 50 μM concentration in water pH 4.0 in the presence of 150 mM SDS at different temperatures determined by the Dichroweb server (K2D).
