# Supplementary material for: Key Physicochemical Determinants in the Antimicrobial Peptide RiLK1 Promote Amphipathic Structures
Source: Int J Mol Sci. 2021 Sep 16;22(18):10011. doi: 10.3390/ijms221810011 (PMC8472000; doi:10.3390/ijms221810011)
Supplement: Supplementary file 1 [file ijms-22-10011-s001.zip › Supplementary Tables.pdf]

Table S1. Secondary structure contents of (**A**) RiLK1 and (**B**) 1018-K6 at 50 μM concentration in water pH 4 in presence of different concentrations of SDS determined by Dichroweb server (K2D).

A

	**α-helix**	**β-sheet**	**Random coil**
**SDS 50 mM**	27%	32%	40%
**SDS 150 mM**	27%	33%	40%

B

	**α-helix**	**β-sheet**	**Random coil**
**SDS 50 mM**	0%	100%	0%
**SDS 150 mM**	0%	100%	0%

Table S2. Secondary structure contents of (**A**) RiLK1 and (**B**) 1018-K6 at different concentrations in water pH 4.0 in presence of 50 mM SDS determined by Dichroweb server (K2D).

A

	**α-helix**	**β-sheet**	**Random coil**
**RiLK1 12.5 µM**	8%	46%	46%
**RiLK1 25 µM**	8%	45%	47%
**RiLK1 50 µM**	19%	32%	49%

B

	**α-helix**	**β-sheet**	**Random coil**
**1018-K6 12.5 µM**	35%	15%	50%
**1018-K6 25 µM**	21%	54%	25%
**1018-K6 50 µM**	12%	82%	5%

Table S3. Secondary structure contents of (**A**) 1018-K6 and (**B**) RiLK1 50 µM concentration in water pH 4 in presence of 150 mM SDS at different times determined by Dichroweb server (K2D).

A

	**α-helix**	**β-sheet**	**Random coil**
**t=0 with SDS**	0%	100%	0%
**t=5 min**	0%	100%	0%
**t=10 min**	7%	90%	3%
**t=30 min**	0%	99%	0%
**t=60 min**	8%	89%	3%
**t=90 min**	9%	96%	1%
**t=120 min**	9%	96%	1%
**t=150 min**	7%	90%	3%
**t=180 min**	6%	92%	3%
**t=240 min**	13%	82%	5%
**t=300 min**	14%	80%	6%
**t=360 min**	12%	83%	5%
**t=1320 min**	12%	83%	5%
**t=1440 min**	14%	80%	6%

B

	**α-helix**	**β-sheet**	**Random coil**
**t=0 with SDS**	11%	35%	54%
**t=5 min**	21%	31%	48%
**t=10 min**	9%	42%	49%
**t=30 min**	11%	37%	52%
**t=60 min**	11%	37%	52%
**t=90 min**	13%	34%	53%
**t=120 min**	8%	43%	48%
**t=150 min**	11%	36%	53%
**t=180 min**	22%	30%	48%
**t=240 min**	15%	34%	51%
**t=300 min**	10%	38%	52%
**t=360 min**	21%	31%	48%
**t=1320 min**	26%	33%	41%
**t=1440 min**	25%	32%	43%

Table S4. Secondary structure contents of (**A**) 1018-K6 and (**B**) RiLK1 at 50 µM concentration in water at different pHs in presence of 150 mM SDS determined by Dichroweb server (K2D).

A

		α-helix	β-sheet	Random coil
pH 2.0	t=0 with SDS	9%	87%	4%
	t=24 h	4%	94%	2%
	t=48 h	9%	87%	4%
pH 7.0	t=0 with SDS	0%	100%	0%
	t=24 h	4%	94%	2%
	t=48 h	0%	100%	0%
pH 12.0	t=0 with SDS	79%	0%	21%
	t=24 h	62%	6%	31%
	t=48 h	58%	9%	34%

B

		α-helix	β-sheet	Random coil
pH 2.0	t=0 with SDS	11%	37%	52%
	t=24 h	7%	51%	42%
	t=48 h	8%	46%	47%
pH 7.0	t=0 SDS	7%	51%	42%
	t=24 h	8%	45%	47%
	t=48 h	7%	51%	42%
pH 12.0	t=0 with SDS	10%	35%	55%
	t=24 h	7%	48%	45%
	t=48 h	7%	51%	42%

Table S5. Secondary structure contents of (A) 1018-K6 and (B) RiLK1 at 50 µM concentration in water pH 4.0 in presence of 150 mM SDS at different temperatures determined by Dichroweb server (K2D).

A

		α-helix	β-sheet	Random coil
**4 °C**	**t=0 with SDS**	0%	100%	0%
	**t=24 h**	0%	100%	0%
	**t=48 h**	1%	99%	1%
**37 °C**	**t=0 with SDS**	0%	100%	0%
	**t=24 h**	0%	100%	0%
	**t=48 h**	2%	96%	2%
**90 °C**	**t=0 with SDS**	0%	100%	0%
	**t=24 h**	2%	51%	47%
	**t=48 h**	12%	39%	49%

B

		α-helix	β-sheet	Random coil
**4 °C**	**t=0 with SDS**	8%	44%	48%
	**t=24 h**	7%	51%	42%
	**t=48 h**	8%	45%	47%
**37 °C**	**t=0 with SDS**	8%	44%	48%
	**t=24 h**	7%	51%	42%
	**t=48 h**	7%	48%	44%
**90 °C**	**t=0 with SDS**	8%	44%	48%
	**t=24 h**	7%	51%	42%
	**t=48 h**	7%	50%	42%
