# Supplementary material for: Key Physicochemical Determinants in the Antimicrobial Peptide RiLK1 Promote Amphipathic Structures
Source: Int J Mol Sci. 2021 Sep 16;22(18):10011. doi: 10.3390/ijms221810011 (PMC8472000; doi:10.3390/ijms221810011)
Supplement: Supplementary file 1 [file ijms-22-10011-s001.zip › Figure S2.pdf]

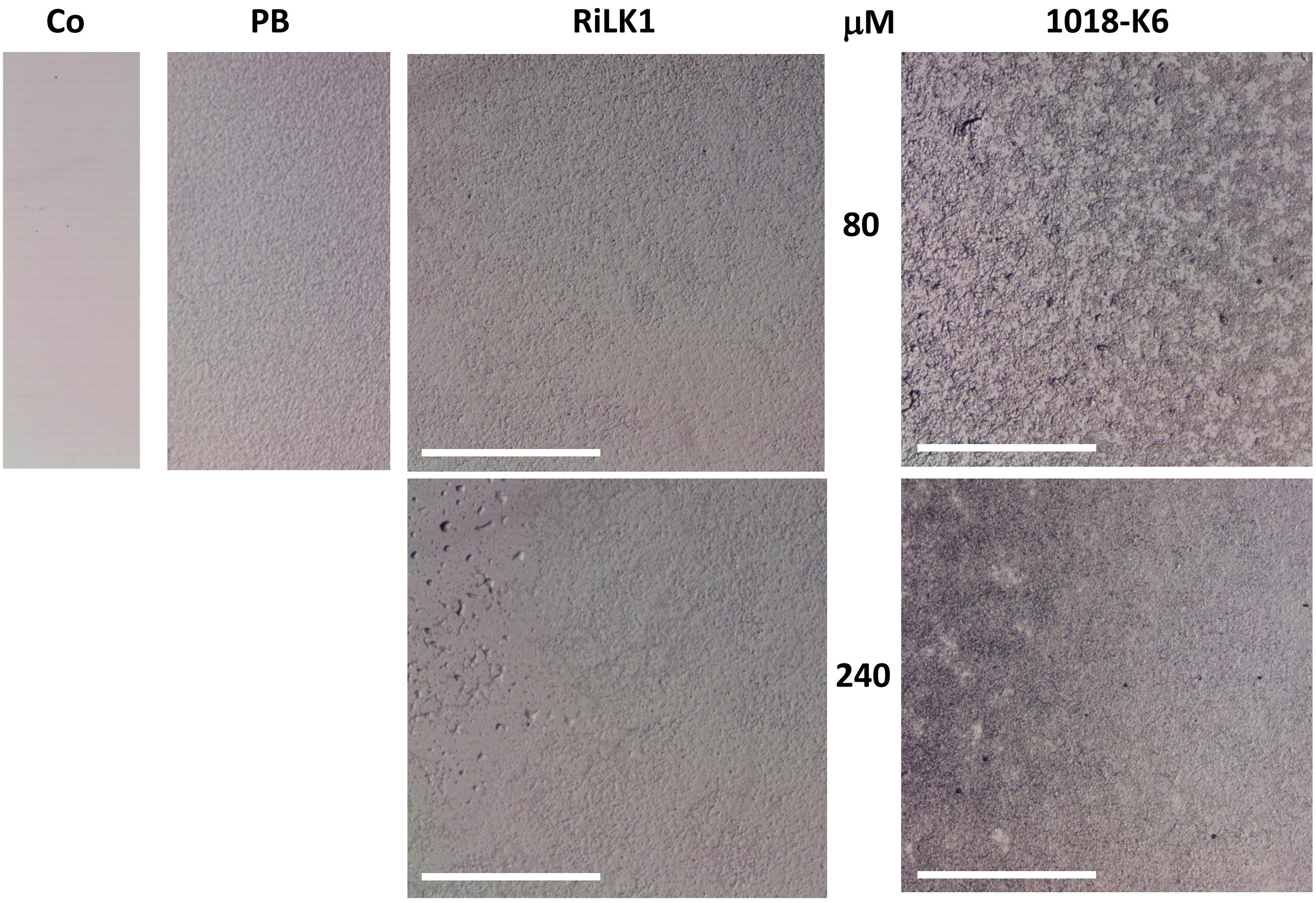

Figure S2. Stereomicroscopic analysis of RILK1 and 1018-K6 in PB. The two peptides were resuspended in phosphate buffer (PB), dried at 60 °C O/N and observed at stereomicroscope Leica MZ16-FA. The plate and PB alone were used as controls. Bar is equal to 1mm.
