# Supplementary material for: Key Physicochemical Determinants in the Antimicrobial Peptide RiLK1 Promote Amphipathic Structures
Source: Int J Mol Sci. 2021 Sep 16;22(18):10011. doi: 10.3390/ijms221810011 (PMC8472000; doi:10.3390/ijms221810011)
Supplement: Supplementary file 1 [file ijms-22-10011-s001.zip › Figure S4.pdf]

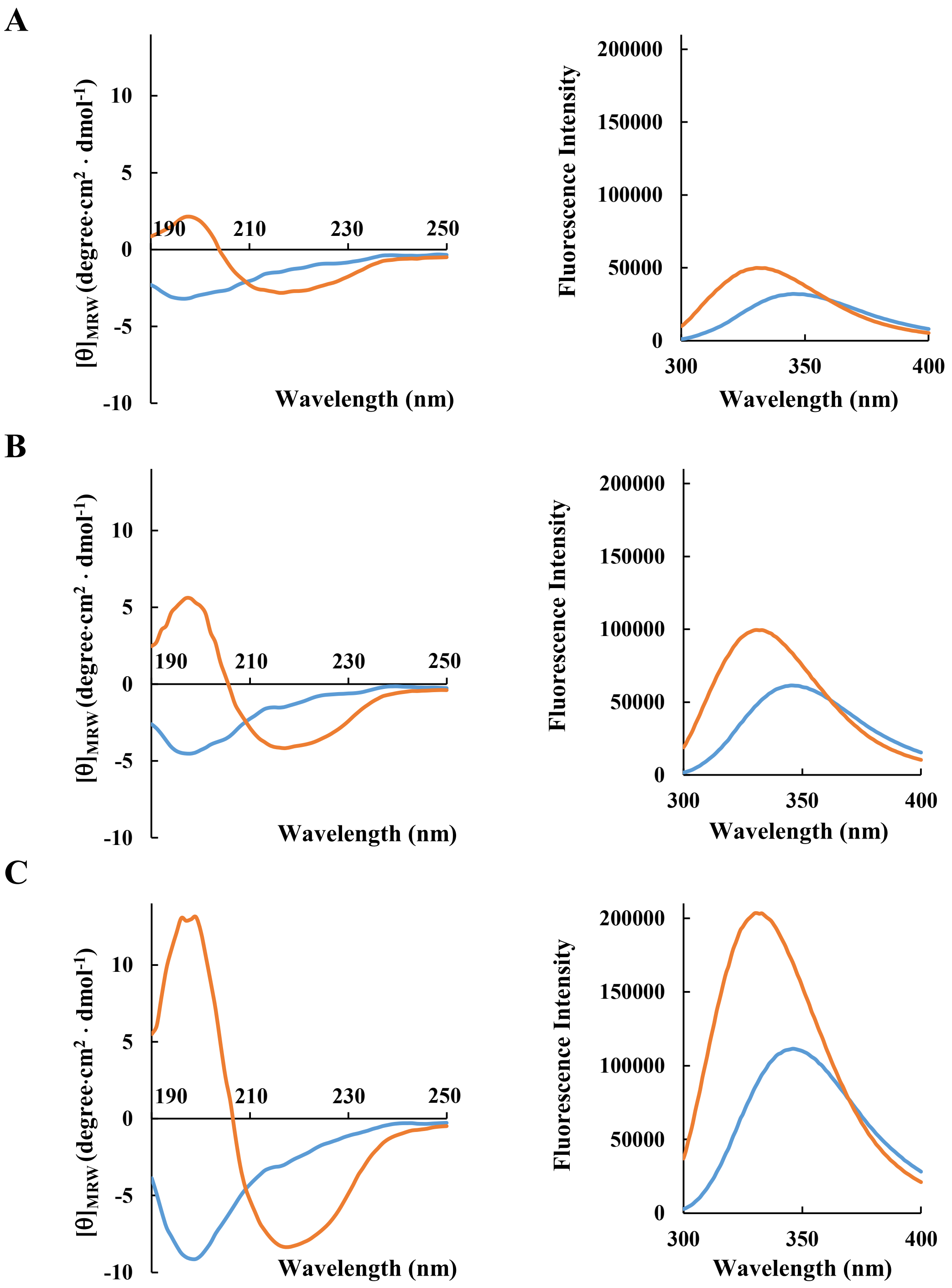

Figure S4. CD (left) and fluorescence (right) spectra of 1018-K6 at increasing peptide concentration. (**A**) 12.5 μM, (**B**) 25 μM and (**C**) 50 μM in water pH 4.0 (blue lines) and with 50 mM SDS (orange lines).
