# Supplementary material for: Key Physicochemical Determinants in the Antimicrobial Peptide RiLK1 Promote Amphipathic Structures
Source: Int J Mol Sci. 2021 Sep 16;22(18):10011. doi: 10.3390/ijms221810011 (PMC8472000; doi:10.3390/ijms221810011)
Supplement: Supplementary file 1 [file ijms-22-10011-s001.zip › Figure S5.pdf]

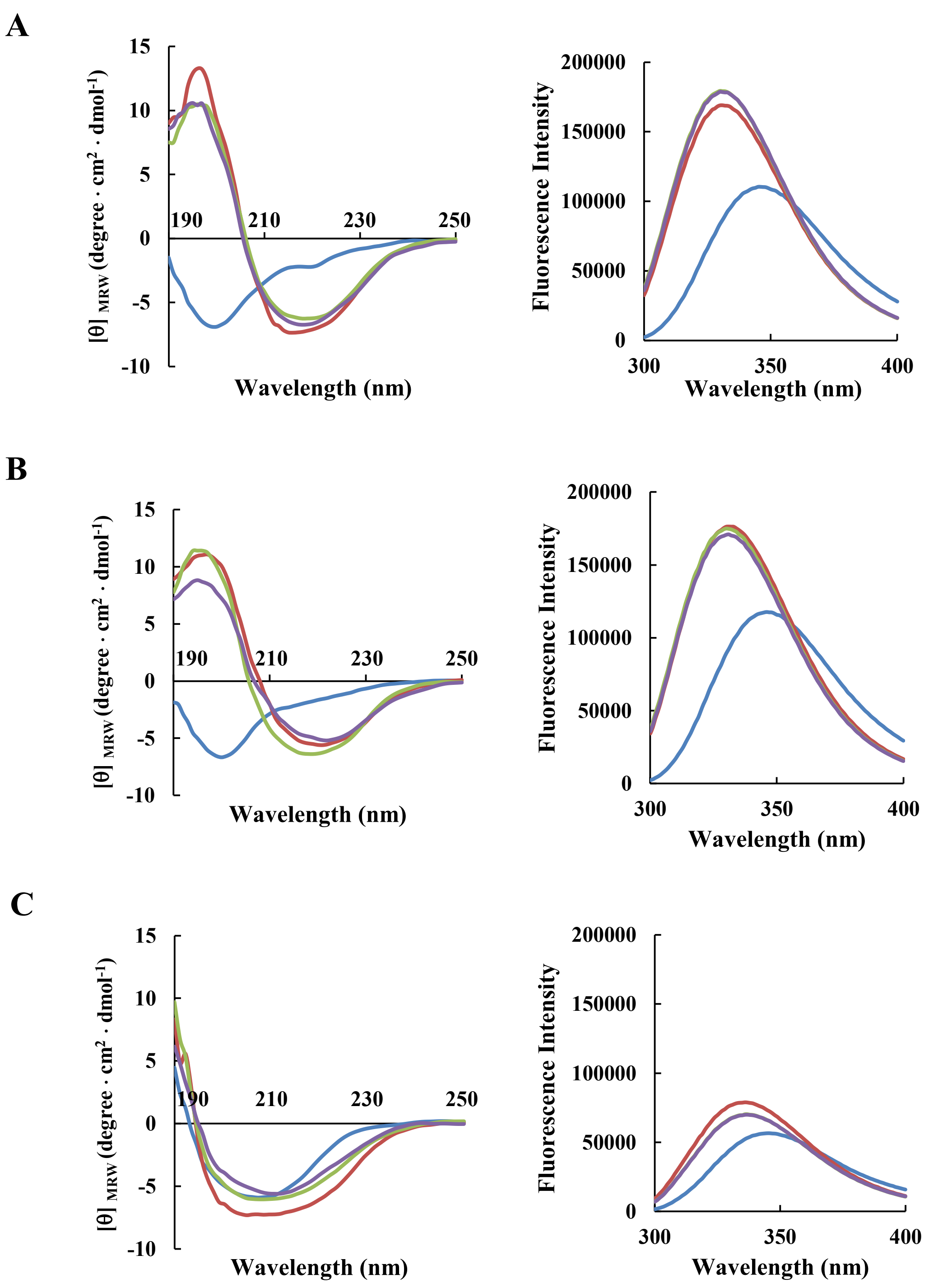

Figure S5. Effect of pH on the folding and secondary structure of 1018-K6 monitored by spectroscopic techniques. CD and fluorescence spectra of 1018-K6 at 50 μM concentration were recorded in water at (**A**) pH 2.0, (**B**) pH 7.0 and (**C**) pH 12.0, in the presence of 150 mM SDS up to 48 h incubations. The spectra of peptide samples in absence of SDS (blue lines) were also reported. The spectra were recorded at t=0 (magenta lines), t=24 h (green lines) or t=48 h (purple lines).
