# Supplementary material for: Key Physicochemical Determinants in the Antimicrobial Peptide RiLK1 Promote Amphipathic Structures
Source: Int J Mol Sci. 2021 Sep 16;22(18):10011. doi: 10.3390/ijms221810011 (PMC8472000; doi:10.3390/ijms221810011)
Supplement: Supplementary file 1 [file ijms-22-10011-s001.zip › Figure S6.pdf]

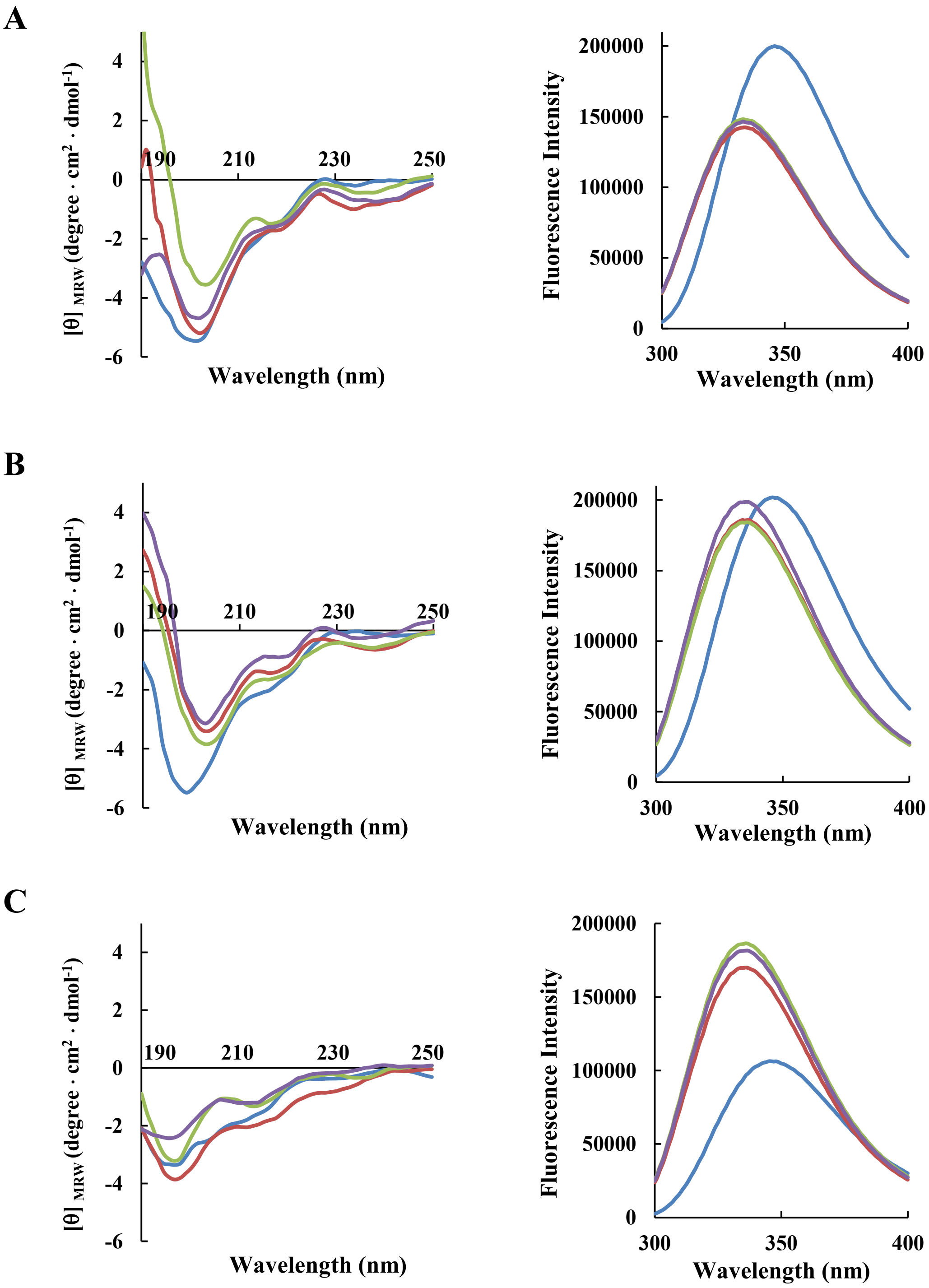

Figure S6. Effect of pH on the folding and secondary structure of RiLK1 monitored by spectroscopic techniques. CD and fluorescence spectra of RiLK1 at 50 μM concentration were recorded in water at (**A**) pH 2.0, (**B**) pH 7.0 and (**C**) pH 12.0, in the presence of 150 mM SDS up to 48 h incubations. The spectra of peptide samples in absence of SDS (blue lines) were also reported. The spectra were recorded at t=0 (magenta lines), t=24 h (green lines) or t=48 h (purple lines).
