# Supplementary material for: Key Physicochemical Determinants in the Antimicrobial Peptide RiLK1 Promote Amphipathic Structures
Source: Int J Mol Sci. 2021 Sep 16;22(18):10011. doi: 10.3390/ijms221810011 (PMC8472000; doi:10.3390/ijms221810011)
Supplement: Supplementary file 1 [file ijms-22-10011-s001.zip › Figure S9.pdf]

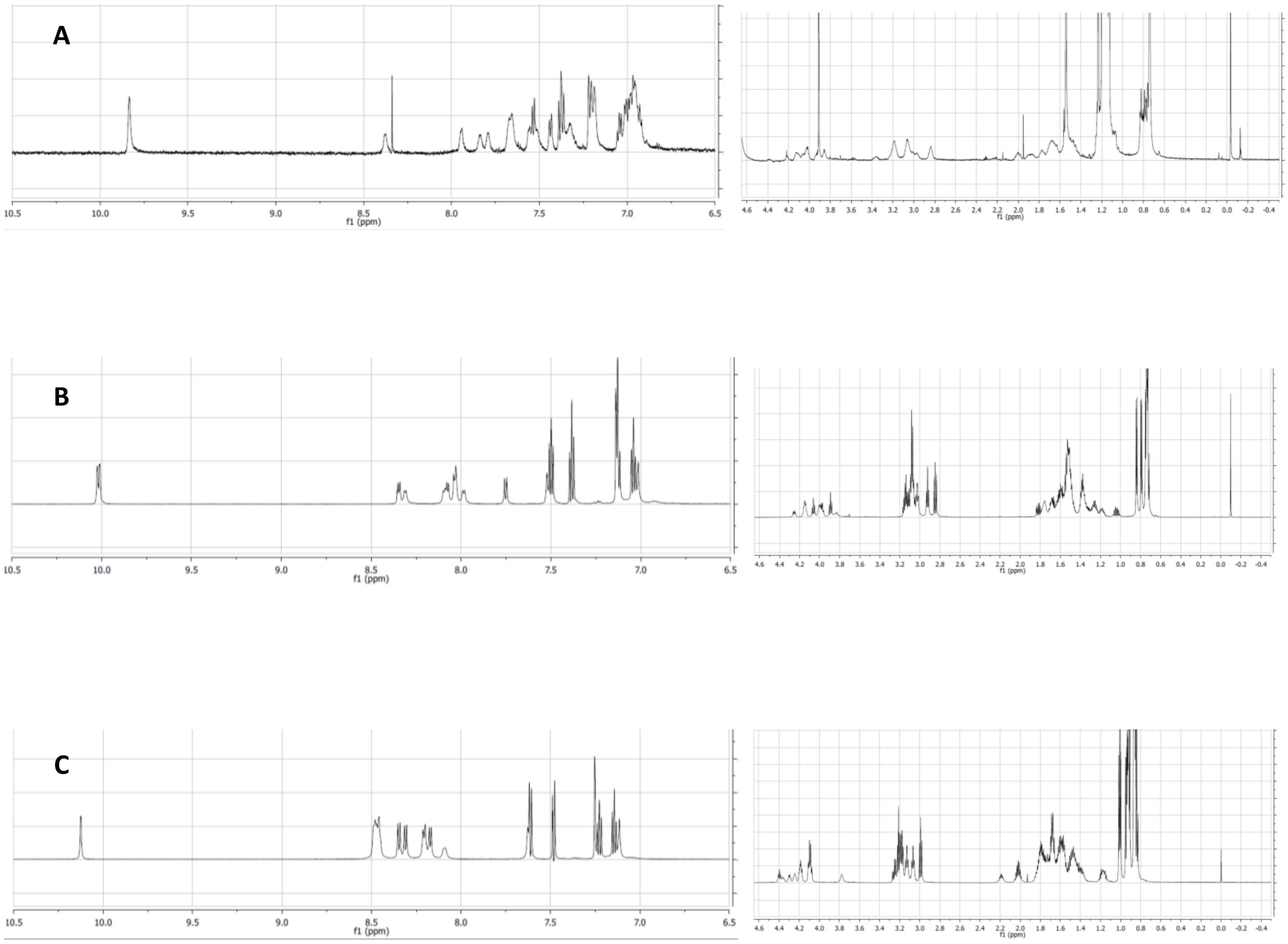

Figure S9. ^{1}H NMR spectra of RiLK1 1.0 mM A) in SDS micelles 150 mM pH 4.4, **B**) in water pH 4; and **C**) of 1018-K6 1.0 mM in water pH 4. Low field (left) and high field regions (right).
