# Supplementary material for: Key Physicochemical Determinants in the Antimicrobial Peptide RiLK1 Promote Amphipathic Structures
Source: Int J Mol Sci. 2021 Sep 16;22(18):10011. doi: 10.3390/ijms221810011 (PMC8472000; doi:10.3390/ijms221810011)
Supplement: Supplementary file 1 [file ijms-22-10011-s001.zip › Figure S10.pdf]

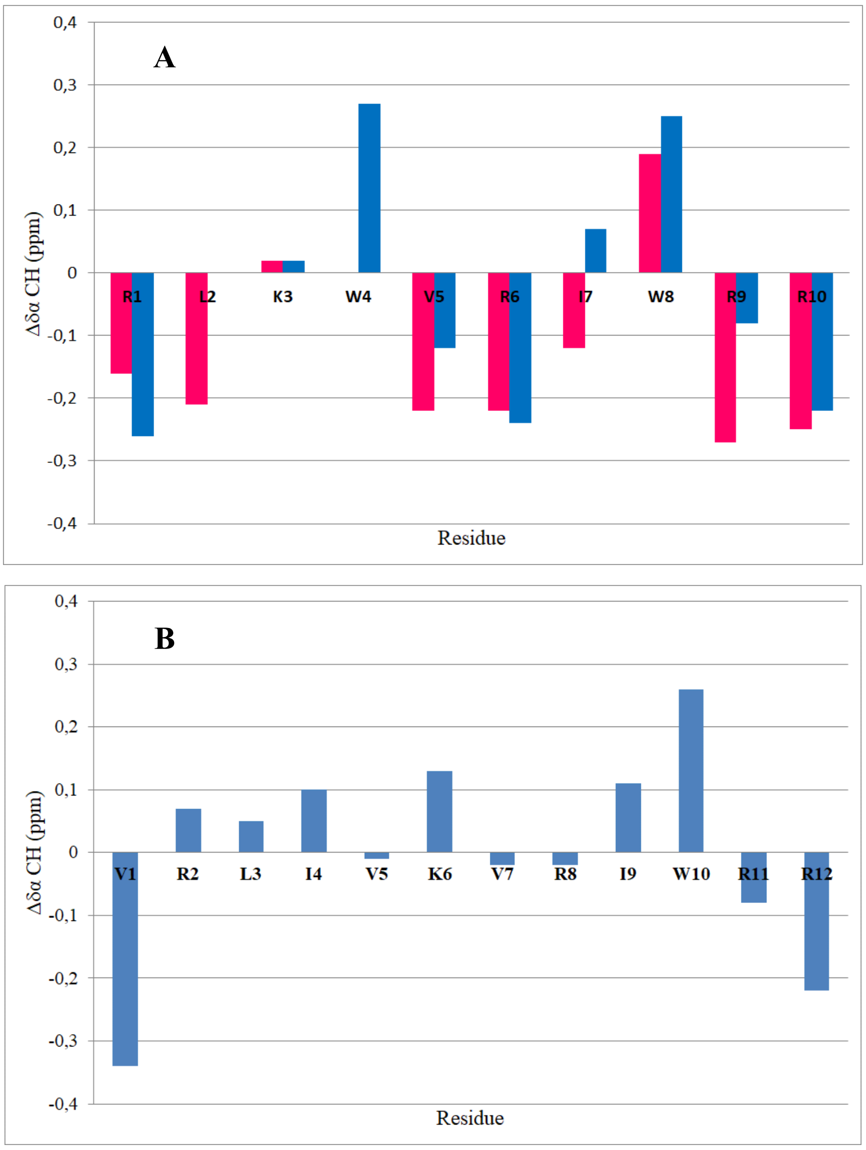

Figure S10. Chemical shift deviations (ppm) from random coil values of aCH protons of **A**) RiLK1 peptide in water (blue bars) and in SDS micelles 150 mM pH 4.4 (fuchsia bars) and **B**) 1018-K6 peptide in water.
