# Supplementary material for: Key Physicochemical Determinants in the Antimicrobial Peptide RiLK1 Promote Amphipathic Structures
Source: Int J Mol Sci. 2021 Sep 16;22(18):10011. doi: 10.3390/ijms221810011 (PMC8472000; doi:10.3390/ijms221810011)
Supplement: Supplementary file 1 [file ijms-22-10011-s001.zip › Figure S11.pdf]

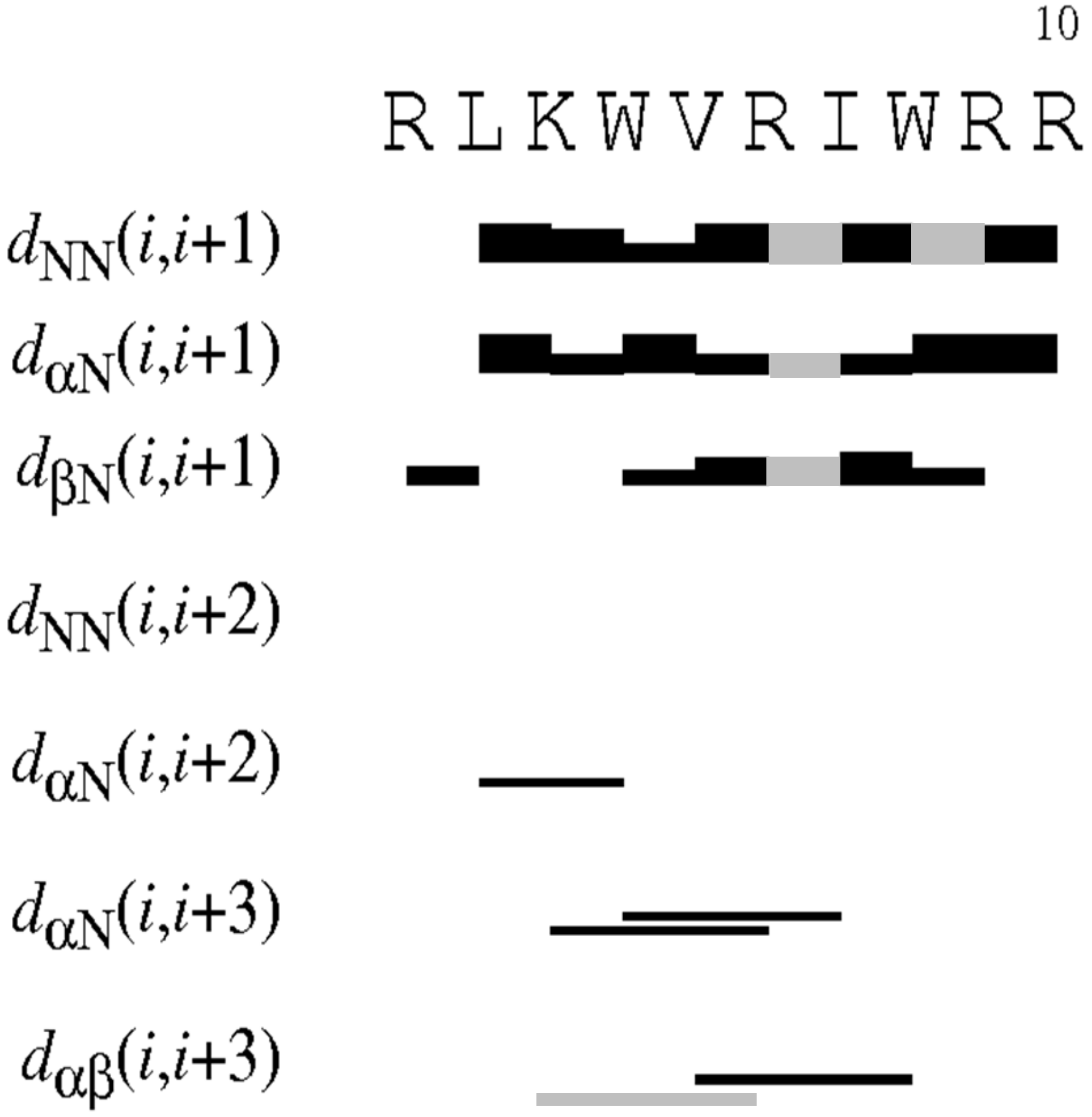

Figure S11. Most relevant NOE effects measured for RiLK1 peptide in SDS. Gray boxes indicate possible NOEs not measured for spectral overlapping and not included as upl in CYANA calculations.
